# Protein-enriched intermittent meal replacement combined with moderate-intensity training for weight loss and body composition in overweight women

**DOI:** 10.1038/s41598-025-96486-6

**Published:** 2025-04-11

**Authors:** Qisijing Liu, Yi Guo, Bo Peng, Dancai Fan, Jing Wu, Jin Wang, Ruican Wang, Jing-Min Liu, Jian Wu, Shuo Wang, Yanrong Zhao

**Affiliations:** 1 Tianjin Key Laboratory of Food Science and Health, School of Medicine, Nankai University, Tianjin, 300071 China; 2 Shanghai M-Action Health Technology Co. Ltd, Shanghai, 201210 China

**Keywords:** IMR, Weight loss, Protein supplementation, Body composition, MICT, Metabolism, Nutrition, Clinical trial design

## Abstract

The global rise in overweight and obesity has been exacerbated by sedentary lifestyles and suboptimal dietary habits. Traditional weight loss methods often struggle with adherence due to restrictive diets and metabolic adaptations. Intermittent meal replacement (IMR), incorporating formulated protein-enriched nutritional shakes, has emerged as a potential strategy for weight management. However, its combined effects with moderate-intensity continuous training (MICT) remain underexplored. This study aimed to evaluate the impact of a weight loss method incorporating formulated protein-enriched nutritional shake IMR in conjunction with MICT workout for overweight female adults. This 8-week parallel randomized controlled trial investigated the impact of protein-enriched IMR combined with MICT on weight loss and body composition in overweight female adults. Participants were randomly assigned to either the MICT group or MICT + IMR group. Body composition, hematological, and urinary biomarkers were assessed pre- and post-intervention. The MICT + IMR Group achieved a greater reduction in body weight (-3.70 kg vs. -1.17 kg, p < 0.001) and body fat mass (-2.25 kg vs. -1.19 kg, p < 0.001) compared to the MICT group. Additionally, fasting blood glucose and insulin levels significantly improved in the MICT + IMR Group, suggesting enhanced metabolic regulation. IMR, when combined with MICT, is a viable strategy for short-term weight loss in overweight women, offering improved fat reduction and metabolic benefits compared to exercise alone.

*Trial registration*: Chinese Clinical Trail Registry, ChiCTR2300076750. Registered 17 October 2023, https://www.chictr.org.cn/bin/project/edit?pid=197611.

**Supplementary Information:**

The online version contains supplementary material available at 10.1038/s41598-025-96486-6.

## Introduction

Obesity and weight management issues have become major global health challenges, affecting billions of people worldwide. Over the past 50 years, the prevalence of overweight and obesity among the global population has risen rapidly^1^. Currently, the overweight and obesity rate among Chinese adults (≥ 18 years old) is approaching 50%, making China one of the countries with the highest number of overweight and obese individuals^2^. Traditionally, Chinese diets included diverse protein sources such as soy products, lean meats, and legumes. However, shifting lifestyles and dietary habits, influenced by work and life pressures^3^, have led to increased consumption of processed and high-carbohydrate foods. This dietary shift may contribute to an imbalance in protein intake, which, coupled with excessive carbohydrate consumption, can exacerbate weight-related issues. Poor dietary habits and sedentary lifestyles further contribute to obesity and its associated health risks, including cardiovascular diseases, type 2 diabetes, hypertension, and even cancer^4–7^.

Conventional weight loss strategies focus on lifestyle modifications, including diet, and exercise, to achieve a negative energy balance. However, strict calorie restriction can be difficult to sustain and may cause nutritional deficiencies, metabolic adaptations, and psychological challenges that hinder long-term success. Weight loss progress often slows over time due to psychological and behavioral factors related to compliance with the intervention^8^. Concurrently, researchers continue to explore alternative and more effective weight loss approaches. In recent years, intermittent energy restriction (IER) has garnered significant attention from researchers and the public as a novel weight loss approach. In comparison to continuous energy restriction, IER involves alternating strategies of energy intake adjustment, potentially offering greater feasibility, sustainability, and sufficient health benefits^9^. Intermittent meal replacement (IMR) is an effective IER method. It was designed to substitute regular meals while ensuring adequate nutrient intake using low-calorie, nutritionally balanced meal replacement products in a intermittent way. This diet can not only help control overall calorie intake in a planned manner without completely fasting, but also provide the body with necessary nutritional support, avoiding discomfort caused by excessive hunger or malnutrition. Common forms of meal replacement (MR) options include beverages, energy bars, and soups, typically providing 200–400 kcal per serving, high-quality proteins, and essential vitamins and minerals. The protein component in the MR formula supports tissue repair, promotes fullness, preserves lean muscle mass, and enhances weight loss and long-term weight management more effectively than very low-calorie diets^10,11^. Some products also incorporate low-energy foods like low-starch vegetables^12,13^, while higher protein intake (≥ 1 g protein/kg body weight) has been shown to aid appetite control and fat-free mass maintenance during weight loss^14^.

Additionally, exercise is undoubtedly the most desirable and important way to lose weight and promote metabolic health^15–19^. It not only increases energy expenditure and fat burning but also enhances metabolism and muscle quality, thus contributing to long-term weight maintenance^20^. In contrast, traditional calorie restriction may lead to a slower weight loss rate and even metabolic adaptations, making the weight loss process more challenging^21^. Therefore, combining protein-enriched nutritional shakes as a IMR approach in conjunction with appropriate exercise might result in synergistic weight loss effects.

Moderate-intensity continuous training (MICT), which involves sustained exercise at 50–70% of maximum heart rate for 20–60 min, is an effective and accessible workout option for overweight individuals^22,23^. Studies indicate that MICT and high-intensity interval training (HIIT) yield similar weight loss outcomes, but MICT is often more sustainable for individuals unaccustomed to intense exercise^24^. Therefore, compared to HIIT, MICT is a much easier yet effective way for overweight people who are not in the habit of exercising. However, research suggests that post-exercise energy intake differs between genders, with women tending to experience increased food consumption, potentially offsetting the caloric deficit created by exercise^25^. Unlike men, women have increased food palatability after exercise and do not observe transient suppression of hunger immediately after exercise^25^. MRs, with controlled calorie content and balanced nutrients, may help mitigate this challenge by providing precise dietary regulation^26^. Additionally, consuming a protein-rich meal after exercise can help minimize fat loss while maintaining muscle mass, a key objective in obesity treatment. Given these considerations, we hypothesize that combining MICT with IMR products may enhance fat loss. This approach leverages the benefits of both exercise and controlled nutrition to optimize weight management outcomes.

Both MR and MICT have independently demonstrated benefits for weight loss and metabolic health^27,28^. Given these findings, our research design specifically aims to investigate the synergistic effects of these interventions. Specifically, we investigate whether replacing dinner with a protein-enriched nutritional shake three nonconsecutive days per week, in conjunction with MICT, can effectively reduce body fat, body weight, and waist circumference in overweight women without significant muscle loss. Additionally, we assess improvements in glycolipid metabolism, which may lower the risk of chronic diseases. By evaluating the safety, feasibility, and effectiveness of this approach, our research seeks to provide evidence-based guidance for personalized weight loss strategies, promoting healthier dietary habits and sustainable weight management.

## Materials and methods

### Participants

The sample size was calculated using PASS software (version 15.0) based on a two-sided two-sample equal variance t-test with a 3 kg between-group weight change difference (approximately 5% for individuals weighing 60 kg) and an SD of 3 kg between the IMR and MICT Groups^29–31^. A total of 17 participants provided 80% power to detect this difference at a two-tailed significance level of 0.05. To account for potential attrition, we recruited 40 participants, assuming a 10% dropout rate.

Forty healthy female adults aged 18–35 from Nankai University were enrolled. Inclusion criteria required a body mass index (BMI) of 24–28 kg/m² (according to the criteria of the Working Group on Obesity in China), body fat percentage ≥ 28%, or a waist-to-hip ratio of ≥ 0.8.

Exclusion criteria included food allergies or aversions to study products, unwillingness to adhere to dietary or exercise protocols, medical conditions unsuitable for exercise, gastrointestinal disorders, thyroid abnormalities, obesity-related chronic diseases (e.g., diabetes, cardiovascular diseases, hypertension, metabolic disorders), concurrent dietary supplement use, smoking, alcohol use, or any other condition deemed inappropriate by the principal investigator.

This study employed a parallel randomized controlled trial design, with participants randomly assigned (1:1) to either the MICT + IMR Group or the MICT group (exercise only). Randomization was stratified by baseline BMI categories (24–26.9 kg/m² and 27–28 kg/m²) to ensure balanced allocation. An independent researcher, uninvolved in recruitment, intervention management, or outcome assessment, generated the random sequence. Group allocation was concealed in sealed opaque envelopes, opened only after participant enrollment.

Recruitment was conducted from February 16, 2023 to March 27, 2023, via online advertisements and local community outreach. Participants were screened based on the predetermined criteria upon voluntary enrollment and provided written informed consent before enrollment. This trial was registered with the Chinese Clinical Trail Registry (ChiCTR2300076750) on 2023-10-17. Ethical approval for this study was obtained from the Nankai University Biomedical Ethics Review Board (NKUIRB2022138).

### Intervention protocol

This trial was an 8-week parallel randomized control trial. Participants in both groups underwent baseline assessments at week 0, followed by a follow-up assessment at week 3, and post-intervention assessment at week 8. During these visits, participants underwent measurements and collection of biological samples.

Both groups selected 3 nonconsecutive intervention days per week, during which they engaged in a 40-minute MICT workout. The MICT + IMR Group followed an IER-based approach, consuming a protein-enriched nutritional shake within 30 min post-exercise as a dinner replacement. In contrast, the MICT Group was instructed to maintain their habitual dinner after excercise.

On non-intervention days, both groups adhered to a calorie-restricted diet. Following the guidelines outlined in the “China Blue Paper on Obesity Prevention and Control,” participants were advised to maintain a well-balanced daily intake of 1,000–1,500 kcal. This approach aimed to prevent excessive consumption of unhealthy, high-calorie foods while minimizing disruption to habitual eating patterns. The MICT workout were supervised by a certified exercise physiologist, with heart rate monitored throughout using Apple Watch Series 8, Apple Inc. which was demonstrated good accuracy for heart rate measurement^32^. The workout protocol consisted of 24 aerobic dance-based training sessions, each lasting approximately 40 min, Sessions included a 5 min warm-up session (50% HRmax), a 30 min continuous aerobic dance loading session (60–70% HRmax), and a 5 min cool-down session (50% HRmax). HRmax was estimated using the age-predicted maximal heart rate (APMHR) equation: HRmax = 220 - Age proposed by Fox et al. in 1971^33^.

### Study outcomes

Outcomes were assessed at baseline and in weeks 3 and 8. These assessments were questionnaire surveys, physical examinations, and collection of blood and urine samples. Primary outcomes included changes in body weight, while secondary outcomes comprised body fat mass and skeletal muscle mass, measured via bioelectrical impedance analysis (InBody 260, Biospace, California, USA) measured at baseline, week 3 and week 8 of the intervention. At baseline and week 8 of the intervention, overnight fasting venous blood and urine were collected to measure metabolic biomarkers, including plasma albumin, glucose, insulin, cholesterol, triglyceride, low-density lipoprotein cholesterol (LDL-C), high-density lipoprotein cholesterol (HDL-C), C-reactive protein (CRP), myeloperoxidase (MPO), malondialdehyde (MDA), as well as liver and kidney function biomarkers, including aspartate aminotransferase (AST), alanine aminotransferase (ALT), bilirubin, and urine ketones. Fasting (> 12 h) venous blood samples were collected by a research nurse, and measurements of the aforementioned biomarkers were performed using clinical chemistry analyzers, colorimetric method (Nanjing Jiancheng), and ELISA (Cusabio).

At baseline and week 8 of the intervention, a 3-day food diary was used to record all foods (meals, snacks, drinks, etc.) consumed by participants on one intervention day, one regular workday (non-intervention day), and one weekend day (non-intervention day). Nutrient intakes from food records were calculated based ib tge data frm the “China Food Composition Tables Standard Edition (6th Edition/Volume 1)” and “China Food Composition Tables (6th Edition/Volume 2)”. Data entry was conducted using EpiData (version 3.1), followed by data cleaning and analysis through STATA (version 17.0) software.

### Study product

The study product was a formulated protein-enriched nutritional shake, donated by Shanghai M-Action Health Technology Co. Ltd. Study products were available in two flavors (original and aloe avocado flavor) and were presented in a 300 mL ready-to-drink (RTD) format. Each bottle of test product provided 200 kcal, 15 g protein, 4.5 g fat, 21.0 g carbohydrate, 6.0 g fiber, and 14 vitamins and minerals. Nutrition details and their comparison with Chinese nutritional reference values are shown in Table 1.

**Table 1:** Nutrition details and comparison of the formulated protein-enriched nutritional shake.

Nutrient component	Per bottle (300 mL)	%NRV
Energy	838 kJ	10%
Protein (Concentrated Milk Protein, Collagen Peptides)	15.0 g	25%
Fat (Coconut Oil)	4.5 g	7.5%
Carbohydrate (Maltodextrin, Starch)	21.0 g	7%
Dietary Fiber (Resistant Dextrin, Fructooligosaccharides (FOS))	6.0 g	24%
Calcium	318 mg	40%
Sodium	60 mg	3%
Phosphorus	180 mg	25.7%
Selenium	10.0 µg	20%
Vitamin C	9.5 mg	9.5%
Vitamin A	100 µg	12.5%
Vitamin E	2.79 mg α-TE	20%
Vitamin D	3.0 µg	60%
Vitamin B1	0.29 mg	20.7%
Vitamin B2	0.29 mg	20.7%
Vitamin B6	0.29 mg	20.7%
Vitamin B12	0.60 µg	25%
Biotin	6.0 µg	20%
Niacin	2.85 mg	20.4%

### Adverse events

During the trial, adverse events such as bloating, gastric discomfort, nausea, and vomiting were documented. In case of persistent discomfort or severe adverse reactions, the investigator would direct the participants to receive necessary medical or clinical treatment and terminate their trial.

### Statistical analysis

Data entry and verification were conducted by two separate investigators. The normality of model residuals was assessed for the collected data. Appropriate statistical methods were applied for analysis based on the nature of the data. Parametric data was evaluated using Student’s t-test, and paired t-test. All statistical tests were two-tailed, with a significance level set at *p* = 0.05. All data processing for this study was performed using the STATA (version 17.0) software.

## Results

### Baseline characteristics of study participants

**Fig. 1:**
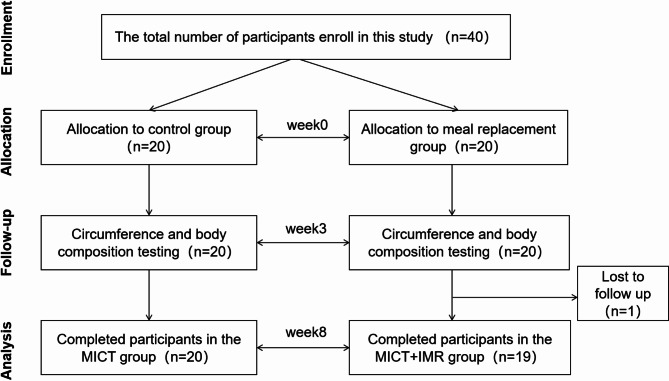
CONSORT diagram of the study.

Initially, 20 participants were recruited and enrolled in each study group. Among them, all participants in the MICT Group completed the intervention trial. In the MICT + IMR Group, 19 individuals completed the trial, while one participant withdrew for personal reasons and her data were excluded for further analysis (Fig. 1). Participants, aged from 19 to 34 years old and with an average age of 23.5 years old, were recruited. The average BMI was 23.68 kg/m² for the MICT + IMR Group and 23.96 kg/m² for the MICT Group (*p* > 0.05). The average body fat percentage was 33.97% for the MICT + IMR Group and 34.18% for the MICT Group (*p*> 0.05). According to the criteria jointly released by the Working Group on Obesity in China (WGOC), our participants (Asian) were overweight to obese^34^. Before the intervention, there were no significant differences in demographic characteristics, anthropometric measures, or body composition between the two groups, indicating that the study participants were well-balanced and comparable (see Additional file 1, Table S1).

### MICT + IMR group showed stronger improvement in body circumference measurements

In the MICT Group, after 3 weeks of calorie-restricted diet and MICT workout intervention, there was a significant reduction in waist circumference (1.25 cm, *p* = 0.022) and calf circumference (0.85 cm, *p* = 0.025) compared with baseline. In the MICT + IMR Group, after 3 weeks of protein-enriched nutritional shakes and MICT workout interventions, there was a significant reduction of 2.32 cm in waist circumference (*p* = 0.004), 1.21 cm in hip circumference (*p* = 0.015), 0.87 cm in upper arm circumference (*p* = 0.033), 0.91 cm in thigh circumference (*p* = 0.030), and 0.68 cm in calf circumference (*p* = 0.013) compared with baseline. Additionally, the waist-to-hip ratio decreased significantly by 0.02% (*p* = 0.026) in the MICT + IMR Group compared with the baseline. However, no significant differences were found in circumference changes (Δ_baseline-followup_) between the two groups (see Additional file 1, Table S2).

After 8 weeks of intervention, the MICT Group exhibited significant reductions of 2.52 cm in waist circumference (*p* = 0.001), 2.16 cm in thigh circumference (*p* = 0.005), 0.97 cm in calf circumference (*p* = 0.026), and a significant decrease of 0.02% in waist-to-hip ratio (*p* = 0.007) compared with baseline. In the MICT + IMR Group, after 8 weeks of protein-enriched nutritional shake and MICT workout interventions, all body circumferences decreased significantly compared with baseline. Notably, chest circumference decreased by 2.46 cm (*p* = 0.001), waist circumference by 4.93 cm (*p* < 0.001), hip circumference by 3.26 cm (*p* < 0.001), upper arm circumference by 2.48 cm (*p* < 0.001), thigh circumference by 2.94 cm (*p* < 0.001), and calf circumference by 1.75 cm (*p* < 0.001). Additionally, the waist-to-hip ratio decreased significantly by 0.03% (*p* = 0.002). Moreover, significant differences were observed in changes in chest circumference, waist circumference, hip circumference, and upper arm circumference (Δ_baseline-followup_) between the two groups (see Additional file 1, Table S2).

These results indicate that incorporating the protein-enriched nutritional shake into the weight loss regimen has a significant effect on decreasing body circumference measurements, particularly in the trunk area (chest circumference, waist circumference, hip circumference) and upper arm circumference, showing significant improvements compared with the MICT Group.

### MICT + IMR group showed stronger improvement in body weight and body composition

At week 3, there was a significant decrease of 0.57 kg in body weight (*p* = 0.022), a reduction of 0.94 kg in body fat (*p* < 0.001), an increase of 0.27 kg in skeletal muscle mass (*p* = 0.036), a decrease of 0.23 kg/m^2^ in BMI (*p* = 0.016), a decrease of 1.20% in body fat percentage (*p* = 0.001), and a significant reduction in body fat mass in limbs and trunk in the MICT Group compared with baseline.

The MICT + IMR Group exhibited a significant decrease in body weight by 2.29 kg (*p* < 0.001) compared with baseline at week 3. Specifically, body fat mass decreased by 1.45 kg (*p* < 0.001), skeletal muscle mass decreased by 0.42 kg (*p* = 0.01), BMI decreased by 0.82 kg/m^2^ (*p* < 0.001), body fat percentage decreased by 1.12% (*p* = 0.04), and body fat mass decreased significantly in various body segments. The MICT + IMR Group exhibited significantly higher reductions in body weight, lean body mass, skeletal muscle mass, and BMI compared with the MICT Group (*p* < 0.05). Both the control and MICT + IMR Groups showed significant reductions in body fat mass across various body segments, with the MICT + IMR Group demonstrating a significantly higher reduction in trunk fat mass, showing statistically significant differences (see Additional file 1, Table S3).

After an 8-week intervention in the MICT Group, we observed a statistically significant reduction in body weight by 1.17 kg (*p* = 0.016), a decrease in body fat mass by 1.19 kg (*p* < 0.001), a decline in BMI by 0.45 kg/m^2^ (*p* = 0.015), and a reduction of 1.24% in body fat percentage (*p* < 0.001) compared with baseline. In addition, body fat mass was significantly reduced in the limbs and trunk of the MICT Group, but lean body mass and skeletal muscle mass did not change significantly. In terms of the MICT + IMR Group, there was a significant decrease in body weight by 3.70 kg (*p* < 0.001), predominantly attributable to a reduction in body fat mass by 2.25 kg (*p* < 0.001) and a gentle decrease in skeletal muscle mass by 0.83 kg (*p* < 0.001) compared with baseline. A significant reduction of BMI by 1.35 kg/m^2^ (*p* < 0.001) and a decline of 1.78% in body fat percentage (*p* = 0.008) were also observed. Further analysis revealed significant reductions in body fat mass in limbs and trunk area within the MICT + IMR Group. Notably, there was a loss of upper limb and trunk muscle mass in the MICT + IMR Group. Upon completion of the 8-week intervention, the MICT + IMR Group demonstrated a markedly higher degree of reduction in body weight, lean body mass, skeletal muscle mass, BMI, and body fat mass compared with the MICT Group (*p* < 0.001). However, while achieving body fat reduction, there was a concomitant moderate loss in skeletal muscle mass in the MICT + IMR Group (Table 2).

**Table 2:** Changes in body composition before and after interventions for 8 week.

Variable	MICT Group				MICT + IMR Group				*p*_delta_
	Baseline	Post-intervention	Δbaseline-post	*p*	Baseline	Post-intervention	Δbaseline-post	*p*	
Height (cm)	162.33(5.66)	162.33(5.66)	–	–	164.90(5.53)	164.89 (5.53)	–	–	-
Age (y)	23.35 (4.32)	23.55 (4.45)	−0.20	0.042	23.53 (4.12)	23.63 (4.14)	−0.11	0.163	0.426
Weight (kg)	63.21 (7.99)	62.04 (8.66)	1.17	0.016	64.39 (7.74)	60.69 (7.97)	3.70	< 0.001	< 0.001
Total Body Water (L)	30.31 (3.33)	30.33 (3.78)	−0.02	0.942	30.95 (3.00)	29.91 (2.96)	1.04	0.001	0.002
Protein (kg)	8.14 (0.90)	8.15 (1.06)	−0.01	0.935	8.32 (0.80)	8.01 (0.81)	0.31	< 0.001	0.001
Inorganic Salt (kg)	3.03 (0.36)	3.02 (0.38)	0.01	0.659	3.09 (0.33)	2.97 (0.31)	0.12	0.001	0.009
Body Fat Mass (kg)	21.75 (4.58)	20.56 (4.77)	1.19	< 0.001	22.04 (4.54)	19.79 (5.16)	2.25	< 0.001	0.035
Lean Body Mass (kg)	41.47 (4.56)	41.48 (5.21)	−0.02	0.958	42.35 (4.10)	40.91 (4.06)	1.45	< 0.001	0.002
Skeletal muscle Mass (kg)	22.53 (2.72)	22.57 (3.15)	−0.05	0.800	23.06 (2.42)	22.23 (2.43)	0.83	< 0.001	0.001
BMI (kg/m^2^)	23.96 (2.47)	23.51 (2.63)	0.45	0.015	23.68 (2.75)	22.33 (2.77)	1.35	< 0.001	< 0.001
Body Fat Percentage (%)	34.18 (3.87)	32.95 (4.07)	1.24	< 0.001	33.97 (3.58)	32.19 (4.89)	1.78	0.008	0.400
Right Upper Limb Muscle (kg)	1.95 (0.31)	1.93 (0.38)	0.01	0.647	2.02 (0.32)	1.85 (0.32)	0.17	< 0.001	0.001
Left Upper Limb Muscle (kg)	1.92 (0.32)	1.89 (0.37)	0.03	0.308	1.97 (0.30)	1.81 (0.31)	0.16	< 0.001	0.001
Trunk Muscle (kg)	18.04 (2.05)	17.93 (2.38)	0.11	0.456	18.55 (1.87)	17.65 (1.97)	0.90	< 0.001	0.175
Right Lower Limb Muscle (kg)	6.45 (0.86)	6.46 (0.95)	−0.02	0.728	6.71 (0.72)	6.64 (0.73)	0.07	0.110	0.087
Left Lower Limb Muscle (kg)	6.39 (0.84)	6.43 (0.96)	−0.04	0.433	6.72 (0.75)	6.64 (0.75)	0.07	0.079	< 0.001
Right Upper Limb Fat (kg)	1.55 (0.43)	1.45 (0.43)	0.10	0.001	1.57 (0.42)	1.39 (0.44)	0.18	< 0.001	0.054
Left Upper Limb Fat (kg)	1.58 (0.41)	1.47 (0.45)	0.11	< 0.001	1.61 (0.41)	1.42 (0.45)	0.19	< 0.001	0.062
Trunk Fat (kg)	10.65 (2.31)	10.02 (2.42)	0.64	< 0.001	10.85 (2.50)	9.46 (2.79)	1.39	< 0.001	0.524
Right Lower Limb Fat (kg)	3.46 (0.72)	3.29 (0.76)	0.17	0.006	3.47 (0.60)	3.25 (0.71)	0.22	0.005	0.690
Left Lower Limb Fat (kg)	3.44 (0.70)	3.27 (0.75)	0.17	0.007	3.45 (0.59)	3.24 (0.71)	0.21	0.007	0.002

Data presented in the form of mean (standard deviation) for all normally distributed variables.

### Biochemical measurements

Obesity has been described as a state of chronic or low-grade systemic inflammation. Weight loss not only improves dyslipidemia and insulin resistance but may also reduce concentrations of inflammation and oxidative stress biomarkers. Following the 8-week intervention, there were no significant changes in insulin and glucose levels for the MICT Group, whereas the insulin and fasting blood glucose levels of the MICT + IMR Group significantly reduced. Both groups exhibited a significant reduction in MPO levels post-intervention compared with baseline, but no group differences were found. No physiological relevant changes in levels of biomarkers such as TG, TC, and CRP were observed either between time points or between two groups (Table 3).

Along with weight loss, levels of the bone formation marker, PICP, significantly decreased, while β-CTx levels significantly increased, with no significant change observed in PINP levels compared with baseline. The decrease in PICP levels indicates a reduction in the ability of bone cells to synthesize collagen, while the increase in β-CTX suggests an elevated rate of bone matrix degradation, indicating increased bone resorption.

No adverse events were reported throughout the course of this study. We observed that after 8 weeks of intervention, 63.16% of participants in the MICT + IMR Group exhibited elevated urinary ketone levels, yet without any instances of ketoacidosis (exceeding 20 mmol/L). This was possibly due to energy and carbohydrate restriction that led to increased fat breakdown and nutritional ketosis. Furthermore, there was no evidence indicating any harm to liver and kidney function due to the intervention. During the intervention period, all biomarkers remained within the normal range, with albumin (ALB) exceeding 35 g/L indicating the absence of malnutrition, therefore the intervention in this study was safe (Table 3).

**Table 3:** Biochemical measurements at baseline and after 8 weeks of intervention.

	MICT group			MICT + IMR group		
	Baseline	Post-intervention	*p*	Baseline	Post-intervention	*p*
Urinary pH value	6.03 (0.57)	6.05 (0.54)	0.863	5.95 (0.33)	5.95 (0.28)	1
Total bilirubin (TBIL) µmol/L	9.52 (3.51)	10.18 (4.96)	0.562	10.02 (4.62)	14.40 (7.11)	0.04
Direct bilirubin (DBIL) µmol/L	4.20 (1.34)	4.68 (1.83)	0.214	4.23 (1.37)	6.49 (2.64)	0.003
Aspartate aminotransferase (AST) U/L	18.73 (2.93)	19.70 (11.39)	0.716	17.81 (3.84)	17.37 (4.54)	0.755
AST/alanine aminotransferase (S/L)	1.66 (0.49)	1.70 (0.54)	0.75	1.78 (0.42)	1.67 (0.37)	0.414
Alanine aminotransferase (ALT) U/L	12.57 (4.98)	11.65 (3.54)	0.442	10.77 (4.09)	11.58 (7.30)	0.71
Total protein (TP) g/L	72.04 (2.87)	72.31 (3.37)	0.664	71.93 (3.06)	74.97 (3.72)	0.001
Albumin (ALB) g/L	49.13 (2.24)	47.81 (2.16)	0.015	48.52 (2.28)	48.68 (1.75)	0.747
High-density lipoprotein cholesterol (HDL-CH) mmol/L	1.45 (0.36)	1.50 (0.334)	0.235	1.55 (0.31)	1.56 (0.39)	0.936
Triglycerides (TG) mmol/L	0.89 (0.42)	0.82 (0.49)	0.292	0.71 (0.18)	0.67 (0.23)	0.478
Total cholesterol (CHOL) mmol/L	4.20 (0.51)	4.08 (0.55)	0.19	4.41 (0.74)	4.41 (0.78)	0.979
Low-density lipoprotein cholesterol (LDL-CH) mmol/L	2.61 (0.46)	2.39 (0.52)	0.007	2.77 (0.61)	2.74 (0.62)	0.861
Glucose (GLU) mmol/L	4.08 (0.19)	4.13 (0.39)	0.583	4.32 (0.32)	3.90 (0.50)	0.003
Insulin (Insulin) µU/L	13.53 (5.52)	13.83 (12.86)	0.926	11.63 (3.74)	7.63 (3.92)	0.005
C-reactive protein (CRP) mg/L	0.92 (1.20)	1.19 (1.12)	0.406	0.75 (0.70)	1.59 (2.30)	0.127
Malondialdehyde (MDA) nmol/mL	2.86 (0.57)	3.40 (1.32)	0.117	2.44 (0.83)	2.43 (2.01)	0.993
Myeloperoxidase (MPO) ng/mL	121.51(88.08)	66.43 (31.81)	0.012	141.55(77.93)	77.77 (29.19)	0.003
β-CTx (β-collagen telopeptide) ng/mL	0.29 (0.12)	0.37 (0.14)	0.002	0.29 (0.15)	0.35 (0.12)	0.027
PICP (Procollagen type I C-terminal propeptide) ng/mL	0.43 (0.19)	0.29 (0.12)	< 0.001	0.43 (0.14)	0.26 (0.09)	< 0.001
PINP (Procollagen type I N-terminal propeptide) ng/mL	63.98 (37.84)	65.41 (31.81)	0.876	55.52 (20.28)	54.82 (16.48)	0.848

Data presented in the form of a mean (standard deviation) for all normally distributed variables.

### Changes in nutrient intake

The total daily calorie intake did not exceed 1500 kcal in both groups, indicating participants had complied with the intervention protocol. At baseline, there were no significant differences in macronutrient intake levels at dinner and total day between the control and MICT + IMR Groups. During the intervention, the MICT + IMR Group exhibited significantly lower total daily intake of energy (IMR: 1058.82 kcal; Control:1390.83 kcal), carbohydrate (IMR: 127.78 g; Control: 164.97 g), fat (IMR: 38.85 g; Control: 58.09 g), and protein (IMR: 49.53 g; Control: 52.05 g) compared with the MICT Group. Especially on intervention day, the MICT + IMR Group exhibited significantly lower total daily and dinner energy intake compared with the MICT Group, but the total day protein intake was not significantly different from that of the MICT Group (Table 4).

During the intervention period, the dietary pattern of the MICT + IMR Group shifted towards a low-energy, protein-enriched, low-fat, and carbohydrate diet with a significant increase in protein energy ratio and decrease in fat energy ratio at dinner and throughout the day compared with baseline. During the intervention period, there were no significant differences in carbohydrate energy ratio between the two groups throughout the day, but the protein energy ratio (19.0%) of the MICT + IMR Group was significantly higher than that of the MICT Group (15.0%). Dietary intakes of the majority of micronutrients were similar between time points and groups (Table 4, Table S4). Additionally, on the intervention day protein energy ratio in the MICT + IMR Group reached 22% compared with that of the MICT Group at 15% (Table 4, Table S5).

**Table 4:** Nutrient intake of the two study groups before and after intervention.

Nutrients	Baseline			Intervention Period			Intervention Day		
	MICT Group	MICT + IMR Group	*p*	MICT Group	MICT + IMR Group	*p*	MICT Group	MICT + IMR Group	*p*
**Macronutrient and energy intake**									
Dinner - Energy (kcal)	407.45 (123.85)	401.75 (165.11)	0.903	518.78 (105.19)	263.76 (112.89)	< 0.001	518.78 (105.19)	200.00 (0.00)	< 0.001
Dinner - Carbohydrates (g)	46.74 (13.19)	47.37 (19.06)	0.904	63.54 (18.66)	31.01 (16.59)	< 0.001	63.54 (18.66)	21.00 (0.00)	< 0.001
Dinner - Fat (g)	16.58 (7.12)	15.74 (9.92)	0.762	19.56 (7.78)	9.23 (5.23)	< 0.001	19.56 (7.78)	4.50 (0.00)	< 0.001
Dinner - Protein (g)	17.82 (7.98)	17.66 (7.49)	0.949	22.16 (8.06)	14.16 (5.73)	0.001	22.16 (8.06)	15.00 (0.00)	0.001
Total Day - Energy (kcal)	1400.17 (363.78)	1461.57 (479.25)	0.654	1390.84 (233.27)	1058.82 (307.62)	0.001	1390.84 (233.27)	910.32 (262.30)	< 0.001
Total Day - Carbohydrates (g)	160.03 (44.33)	194.58 (74.06)	0.084	164.97 (26.48)	127.78 (43.06)	0.003	164.97 (26.48)	108.20 (46.99)	< 0.001
Total Day - Fat (g)	60.38 (19.83)	49.07 (18.16)	0.072	58.09 (14.47)	38.85 (16.85)	0.001	58.09 (14.47)	31.34 (18.95)	< 0.001
Total Day - Protein (g)	54.16 (20.31)	60.40 (18.76)	0.326	52.05 (15.24)	49.53 (13.69)	0.591	52.05 (15.24)	51.04 (22.95)	0.872
**Macronutrient energy ratios (%)**									
Dinner - Carbohydrates	47%	49%	0.686	49%	46%	0.391	49%	42%	0.150
Dinner - Fat	36%	33%	0.415	34%	30%	0.299	34%	20%	< 0.001
Dinner - Protein	17%	18%	0.608	17%	23%	0.006	17%	30%	< 0.001
Total Day - Carbohydrates	46%	53%	0.005	47%	49%	0.709	47%	48%	0.976
Total Day - Fat	39%	30%	< 0.001	38%	33%	0.024	38%	31%	0.002
Total Day - Protein	15%	17%	0.122	15%	19%	0.001	15%	22%	< 0.001
**Other nutrients intake**									
Dietary Fiber (g)	6.57 (2.89)	5.48 (2.01)	0.183	6.82 (3.13)	6.64 (2.99)	0.856	6.82 (3.13)	9.10 (2.82)	0.022
Vitamin D (µg)	1.72 (0.86)	1.69 (1.58)	0.940	1.92 (1.44)	2.07 (1.02)	0.713	1.92 (1.44)	3.70 (0.82)	< 0.001
Vitamin B12 (µg)	1.18 (0.68)	0.39 (0.20)	< 0.001	1.44 (1.28)	0.30 (0.16)	0.001	1.44 (1.28)	0.41 (0.20)	0.002
Vitamin B6 (mg)	0.38 (0.20)	0.87 (0.40)	< 0.001	0.44 (0.23)	0.76 (0.40)	0.004	0.44 (0.23)	1.06 (0.53)	< 0.001
Calcium (mg)	280.96 (100.35)	226.33 (101.44)	0.100	321.17 (164.13)	272.68 (156.64)	0.352	321.17 (164.13)	362.74 (184.21)	0.461
Iron (mg)	11.11 (4.28)	8.73 (2.93)	0.051	10.38 (4.00)	5.96 (2.60)	< 0.001	10.38 (4.00)	4.14 (2.92)	< 0.001
Potassium (mg)	1127.74 (347.14)	1074.82 (338.72)	0.633	1196.78 (373.62)	712.02 (367.43)	< 0.001	1196.78 (373.62)	506.02 (353.92)	< 0.001
Sodium (mg)	2290.10 (860.35)	2265.73 (1268.28)	0.944	2569.87 (1099.02)	1831.75 (1306.34)	0.064	2569.87 (1099.02)	1301.99 (789.27)	< 0.001
Purines (mg)	135.42 (64.67)	119.21 (60.76)	0.426	155.66 (81.13)	87.42 (48.89)	0.003	155.66 (81.13)	65.20 (51.22)	< 0.001
Saturated Fat (g)	7.78 (3.67)	6.68 (2.97)	0.313	7.64 (3.82)	3.79 (2.65)	0.001	7.64 (3.82)	2.09 (2.13)	< 0.001
Sugar (g)	12.68 (8.14)	13.60 (8.41)	0.733	10.25 (6.14)	7.14 (6.09)	0.122	10.25 (6.14)	7.42 (12.61)	0.376

Data presented in the form of mean (standard deviation) for all normally distributed variable.

## Discussion

Obesity has evolved into a global health crisis in China. Achieving weight loss goals through dietary modification and physical activity is considered the healthiest way to reduce weight. Although an 8-week MICT workout (3 times per week) can lead to successive weight loss, our results demonstrated that IMR with a protein-enriched nutritional shake after a MICT workout for 3 days/week for 8 consecutive weeks led to an additional weight loss of 2.53 kg with additional metabolic improvements. These findings are consistent with similar discoveries in previous international, multicenter randomized controlled trial research^35^.

The effectiveness of protein-enrich IMR interventions combined with MICT workout over MICT alone for weight loss can be attributed to several factors. Differences in dietary energy intake and macronutrient composition during the intervention period may be one of the reasons. With no significant differences in carbohydrate intake and MICT workout, IMR interventions with protein-enriched nutritional shakes were characterized by increased protein intake (23% of energy intake) and decreased fat intake (30% of energy intake). This may potentially promote satiety, enhance adherence to dietary requirements, and trigger an elevation in diet-induced thermogenesis. High protein IMR due to their lower calorie content, can replace high-energy meals or snacks, thereby reducing overall energy intake, which significantly impact weight loss^30^. Protein IMRs can also provide balanced nutrition, ensuring an adequate nutrient supply while suppressing excessive calorie intake. Additionally, protein IMRs can assist in controlling carbohydrate intake. Asian diets often contain refined carbohydrates, saturated fats, trans fats, sodium, and sugar, with relatively lower fiber and protein content. By limiting carbohydrate intake, protein-enriched nutritional shakes may help regulate blood sugar and insulin levels, thus contributing to weight loss^36,37^. Lastly, the study product provided good sources of protein, such as concentrated milk protein (mainly composed of whey protein and casein) and collagen peptide, which help maintain muscle mass and increase basal metabolic rate.

Comparatively, the combination of IER and protein-enriched nutritional shake IMR offers distinct advantages in weight loss compared with conventional low-calorie diets. One prominent challenge of adopting a strictly low-calorie diet is the lack of satiety, leading to increased food cravings. Although calorie control can be effective in reducing body weight while improving health, calorie restriction-induced biological adaptations and activation of reward pathways exacerbate hunger and food cravings, ultimately leading to to dietary noncompliance^38,39^. In contrast, protein-enriched IMRs, when combined with MICT workout, offer multiple potential benefits, including enhanced glycemic control, harmonization of nutrient balances, modification of energy intake, control of carbohydrate regulation, optimization of protein supply, improved satiety index, and better feasibility for adherence.

Our results suggest that MICT workout with a protein-enriched IMR interventions induced a reduction in muscle mass, but a more significant effect in fat loss, especially abdominal fat loss, which has a greater benefit in reducing the risk of future metabolic diseases^40,42^. In addition, MICT workout alone showed a plateau in weight loss between weeks 3 and 8, with a significantly less rapid rate of weight loss than in the very first 3 weeks, whereas this was not found in the MICT + IMR Group. Physical adaptation may exist during the MICT workout. Physical exercise provokes an adaptive response over time, leading to a slowdown in weight loss. Protein-enriched IMRs may play an active role in promoting fat loss during exercise weight loss, helping to delaying this plateau. Furthermore, post-exercise protein intake helps to maintain muscle during weight loss.

Despite we found significant improvements in body fat, skeletal muscle, and BMI weight with the protein-enriched IMR intervention compared with the MICT Group, the between-group difference in body fat percentage improvement was not significant, possibly due to the short duration of the intervention. In addition, in the Asian population, the correlation between BMI and body fat percentage is not strong, and BMI is a better predictor of obesity-related biomarkers^43^. This suggests that protein-enriched IMR interventions can be a beneficial strategy to manage overweight and obesity. In addition, insulin and glucose were significantly improved in the MICT + IMR Group than the MICT group, suggesting that is is clinically important as it reflects improved insulin sensitivity and a lower risk of developing type 2 diabetes^44^.while triglycerides and HDL-C showed no significant between-group differences. Previous studies have reported similar findings^30,45,45,46^. Whereas any weight loss strategy aimed at reducing body weight and body fat will be metabolically beneficial, this suggests that short-term protein-enriched IMR interventions are more effective in improving glucose metabolism than lipid metabolism. Future research should explore longer intervention durations and consider metabolic phenotypes, as individuals may respond differently to weight loss interventions depending on gut microbiota and genetic factors.

Notably, long-term use of meal replacements may significantly impact metabolic parameters, warranting caution in practical applications. During the intervention period, we observed a slight increase in bilirubin levels within the normal range, possibly due to weight loss. The depletion of fat stores during the process of weight reduction, releases fatty acids into the bloodstream for metabolism.which may temporarily affect liver function and bilirubin metabolism. However, it was not indicative of abnormal liver function. Additionally, this study demonstrated a reduction in bone formation and an increase in bone resorption due to energy restriction. However, the between-group differences in β-CTx and PICP changes were not significant, indicating that the protein-enriched nutritional shake partially mitigated bone loss associated with rapid weight loss. A decline in skeletal muscle mass may contribute to a reduction in resting energy expenditure (REE), which could affect long-term weight maintenance and metabolic health. This discovery prompts us to pay closer attention to acute bone loss resulting from weight reduction and the importance of protein supplementation. Given that muscle preservation is crucial for maintaining metabolic health, While our protein-enriched IMR may have helped mitigate bone loss, and the differential effects of exercise programs on bone health parameters in individuals with obesity, future research should explore whether adjustments inclusion of resistance training in conjunction with IMR could further enhance muscle retention while reducing weight^47,49^.

This study has certain limitations. A key limitation of this study is the relatively short 8-week intervention period, which may not fully capture the long-term sustainability of weight loss and metabolic improvements. However, previous studies have reported weight changes at varying durations, with a median follow-up of 18 weeks (ranging from 8 to 34 weeks)^27^. This suggests that 8 weeks is a reasonable timeframe for assessing short-term intervention effects, though future research should include longer follow-up periods (e.g., 6–12 months) to evaluate the durability of weight loss and metabolic outcomes.” Secondly, we used food diaries as a way to record dietary energy and nutrients in our research. While reporting bias is an inherent challenge in dietary assessments, food diaries remain one of the most accessible and feasible methods for evaluating dietary intake in free-living settings. Future studies may explore the use of digital tracking tools or biomarkers to complement self-reported data.

## Conclusions

In terms of short-term weight loss, the combination of protein-enriched nutritional shake IMR in conjunction with MICT is a significantly more effective approach compared with the MICT workout alone. The protocol not only enhances the weight management outcomes of pre-obese female adults and facilitates their achievement of weight loss goals, but also has the potential to improve insulin sensitivity and regulate blood glucose levels. However, it is important to emphasize that there is a need for more extensive investigations into the long-term sustainability of these intervention effects and the possible impact on metabolic health.

## Electronic supplementary material

Below is the link to the electronic supplementary material.

Supplementary Material 1

## Data Availability

The original contributions presented in the study are included in the article/supplementary material, further inquiries can be directed to the corresponding author/s.
