# Supplementary material for: Protein-enriched intermittent meal replacement combined with moderate-intensity training for weight loss and body composition in overweight women
Source: Sci Rep. 2025 Apr 11;15:12485. doi: 10.1038/s41598-025-96486-6 (PMC11992100; doi:10.1038/s41598-025-96486-6)
Supplement: Supplementary file 1 — Supplementary Material 1 [file 41598_2025_96486_MOESM1_ESM.docx]

**Protein-enriched Intermittent Meal Replacement Combined with Moderate-Intensity Training for Weight Loss and Body Composition in Overweight Women**

Supplemental Material

Table of Contents：

Table S1：Descriptive analysis of baseline circumference and body composition levels in two groups of participants

Table S2：Changes in body circumference before and after interventions for participants in both groups

Table S3：Changes in body composition before and after interventions for 3 weeks

Table S4: Nutrient intake changes of two study groups before and after intervention

Table S5: Macronutrient energy ratios before and after intervention in two study groups

Table S1**：**Descriptive analysis of baseline circumference and body composition levels in two groups of participants

|  | **Variable** | **MICT Group** | **MICT+MR Group** | ***p*** |
| --- | --- | --- | --- | --- |
| Circumference | Chest (cm) | 90.83 (5.89) | 89.71 (5.80) | 0.555 |
|  | Waist (cm) | 77.83 (8.06) | 75.87 (6.10) | 0.531 |
|  | Hip (cm) | 98.36 (5.86) | 98.26 (5.55) | 0.958 |
|  | Upper arm (cm) | 27.82 (2.23) | 27.71 (2.66) | 0.895 |
|  | Thigh (cm) | 54.37 (4.35) | 55.02 (6.07) | 0.702 |
|  | Calf (cm) | 37.41 (3.08) | 37.30 (2.90) | 0.909 |
|  | Waist-Hip Ratio | 0.79 (0.06) | 0.77 (0.04) | 0.383 |
| Demographic  and  Body Composition | Height (cm) | 162.33 (5.66) | 164.90 (5.53) | 0.160 |
|  | Age (y) | 23.35 (4.32) | 23.53 (4.12) | 0.897 |
|  | Weight (kg) | 63.21 (7.99) | 64.39 (7.74) | 0.642 |
|  | Total Body Water (L) | 30.31 (3.33) | 30.95 (3.00) | 0.531 |
|  | Protein (kg) | 8.14 (0.90) | 8.32 (0.80) | 0.524 |
|  | Inorganic Salt (kg) | 3.03 (0.36) | 3.09 (0.33) | 0.596 |
|  | Body Fat (kg) | 21.75 (4.58) | 22.04 (4.54) | 0.843 |
|  | Lean Body Mass (kg) | 41.47 (4.56) | 42.35 (4.10) | 0.527 |
|  | Skeletal Muscle Mass (kg) | 22.53 (2.72) | 23.06 (2.42) | 0.522 |
|  | BMI (kg/m^2^) | 23.96 (2.47) | 23.68 (2.75) | 0.743 |
|  | Body Fat Percentage (%) | 34.18 (3.87) | 33.97 (3.58) | 0.864 |
|  | Right Upper Limb Muscle (kg) | 1.95 (0.31) | 2.02 (0.32) | 0.484 |
|  | Left Upper Limb Muscle (kg) | 1.92 (0.32) | 1.97 (0.30) | 0.594 |
|  | Trunk Muscle (kg) | 18.04 (2.05) | 18.55 (1.87) | 0.425 |
|  | Right Lower Limb Muscle (kg) | 6.45 (0.86) | 6.71 (0.72) | 0.314 |
|  | Left Lower Limb Muscle (kg) | 6.39 (0.84) | 6.72 (0.75) | 0.206 |
|  | Right Upper Limb Fat (kg) | 1.55 (0.43) | 1.57 (0.42) | 0.862 |
|  | Left Upper Limb Fat (kg) | 1.58 (0.41) | 1.61 (0.41) | 0.849 |
|  | Trunk Fat (kg) | 10.65 (2.31) | 10.85 (2.50) | 0.794 |
|  | Right Lower Limb Fat (kg) | 3.46 (0.72) | 3.47 (0.60) | 0.950 |
|  | Left Lower Limb Fat (kg) | 3.44 (0.70) | 3.45 (0.59) | 0.972 |
|  | Basal Metabolic Rate (kcal) | 1265.70 (98.55) | 1284.74 (88.53) | 0.530 |

Note: data presented in the form of mean (standard deviation) for all normally distributed variables

**Table S2：**Changes in body circumference before and after interventions for participants in both groups

| **Week 3** |  | **Baseline** | **Follow-up** | **Δ_baseline-followup_** | ***p*** | ***p* _delta_** |
| --- | --- | --- | --- | --- | --- | --- |
| MICT Group | Chest (cm) | 90.83 | 91.16 | -0.34 | 0.580 | 0.265 |
|  | Waist (cm) | 77.83 | 76.58 | 1.25 | **0.022** | 0.212 |
|  | Hip (cm) | 98.36 | 97.90 | 0.46 | 0.355 | 0.266 |
|  | Upper arm (cm) | 27.82 | 28.08 | -0.26 | 0.586 | 0.070 |
|  | Thigh (cm) | 54.37 | 53.70 | 0.67 | 0.188 | 0.697 |
|  | Calf (cm) | 37.41 | 36.56 | 0.85 | **0.025** | 0.703 |
|  | Waist-Hip Ratio | 0.79 | 0.78 | 0.01 | 0.137 | 0.558 |
| MICT+MR Group | Chest (cm) | 89.71 | 89.18 | 0.53 | 0.272 |  |
|  | Waist (cm) | 75.87 | 73.55 | 2.32 | **0.004** |  |
|  | Hip (cm) | 98.26 | 97.05 | 1.21 | **0.015** |  |
|  | Upper arm (cm) | 27.71 | 26.84 | 0.87 | **0.033** |  |
|  | Thigh (cm) | 55.02 | 54.11 | 0.91 | **0.030** |  |
|  | Calf (cm) | 37.29 | 36.61 | 0.68 | **0.013** |  |
|  | Waist-Hip Ratio | 0.77 | 0.76 | 0.02 | **0.026** |  |
| **Week 8** |  | **Baseline** | **Post-intervention** | **Δ_baseline-post_** | ***p*** | ***p* _delta_** |
| MICT Group | Chest (cm) | 90.83 | 90.20 | 0.63 | 0.334 | **0.042** |
|  | Waist (cm) | 77.83 | 75.31 | 2.52 | **0.001** | **0.014** |
|  | Hip (cm) | 98.36 | 97.33 | 1.03 | 0.072 | **0.005** |
|  | Upper arm (cm) | 27.82 | 26.91 | 0.91 | 0.144 | **0.043** |
|  | Thigh (cm) | 54.37 | 52.21 | 2.16 | **0.005** | 0.383 |
|  | Calf (cm) | 37.41 | 36.44 | 0.97 | **0.026** | 0.119 |
|  | Waist-Hip Ratio | 0.79 | 0.77 | 0.02 | **0.007** | 0.392 |
| **Week 8** |  | **Baseline** | **Post-intervention** | **Δ_baseline-post_** | ***p*** | ***p* _delta_** |
| MICT+MR Group | Chest (cm) | 89.71 | 87.25 | 2.46 | **0.001** |  |
|  | Waist (cm) | 75.87 | 70.94 | 4.93 | **<0.001** |  |
|  | Hip (cm) | 98.26 | 95.00 | 3.26 | **<0.001** |  |
|  | Upper arm (cm) | 27.71 | 25.23 | 2.48 | **<0.001** |  |
|  | Thigh (cm) | 55.02 | 52.07 | 2.94 | **<0.001** |  |
|  | Calf (cm) | 37.29 | 35.54 | 1.75 | **<0.001** |  |
|  | Waist-Hip Ratio | 0.77 | 0.75 | 0.03 | **0.002** |  |

**Table S3：**Changes in body composition before and after interventions for 3 weeks

| **Variable** | **MICT Group** | | | | **MICT+MR Group** | | | | ***p* _delta_** |
| --- | --- | --- | --- | --- | --- | --- | --- | --- | --- |
|  | **Baseline** | **Follow-up** | **Δ_baseline-followup_** | ***p*** | **Baseline** | **Follow-up** | **Δ_baseline-followup_** | ***p*** |  |
| Height (cm) | 162.33 | 162.33 | - | - | 164.89 | 164.89 | 0.00 | . | - |
| Age (y) | 23.35 | 23.45 | -0.10 | 0.163 | 23.53 | 23.58 | -0.05 | 0.331 | 0.591 |
| Weight (kg) | 63.21 | 62.64 | 0.57 | **0.022** | 64.39 | 62.10 | 2.29 | **<0.001** | **<0.001** |
| Total Body Water (L) | 30.31 | 30.58 | -0.27 | 0.072 | 30.95 | 30.34 | 0.62 | **0.005** | **0.001** |
| Protein (kg) | 8.14 | 8.22 | -0.08 | 0.074 | 8.32 | 8.18 | 0.13 | **0.03** | **0.005** |
| Inorganic Salt (kg) | 3.03 | 3.04 | -0.01 | 0.491 | 3.09 | 2.99 | 0.10 | **<0.001** | **0.001** |
| Body Fat (kg) | 21.75 | 20.81 | 0.94 | **<0.001** | 22.04 | 20.59 | 1.45 | **<0.001** | 0.096 |
| Lean Body Mass (kg) | 41.47 | 41.84 | -0.37 | 0.071 | 42.35 | 41.51 | 0.84 | **0.005** | **0.001** |
| Skeletal Muscle Mass (kg) | 22.53 | 22.79 | -0.27 | **0.036** | 23.06 | 22.64 | 0.42 | **0.012** | **0.001** |
| BMI (kg/m^2^) | 23.96 | 23.73 | 0.23 | **0.016** | 23.68 | 22.86 | 0.82 | **<0.001** | **<0.001** |
| Body Fat Percentage (%) | 34.18 | 32.99 | 1.20 | **0.001** | 33.97 | 32.85 | 1.12 | **0.005** | 0.868 |
| Right Upper Limb Muscle (kg) | 1.95 | 1.96 | -0.01 | 0.67 | 2.02 | 1.92 | 0.09 | **0.001** | **0.003** |
| Left Upper Limb Muscle (kg) | 1.92 | 1.92 | 0.00 | 0.961 | 1.97 | 1.88 | 0.09 | **0.001** | **0.004** |
| Trunk Muscle (kg) | 18.04 | 18.10 | -0.06 | 0.531 | 18.55 | 18.12 | 0.43 | **0.001** | **0.001** |
| Right Lower Limb Muscle (kg) | 6.45 | 6.47 | -0.03 | 0.568 | 6.71 | 6.69 | 0.02 | 0.663 | 0.473 |
| Left Lower Limb Muscle (kg) | 6.39 | 6.44 | -0.05 | 0.233 | 6.72 | 6.70 | 0.02 | 0.525 | 0.182 |
| Right Upper Limb Fat (kg) | 1.55 | 1.47 | 0.09 | **0.001** | 1.57 | 1.45 | 0.13 | **<0.001** | 0.162 |
| Left Upper Limb Fat (kg) | 1.58 | 1.49 | 0.09 | **0.001** | 1.61 | 1.47 | 0.13 | **<0.001** | 0.144 |
| Trunk Fat (kg) | 10.65 | 10.16 | 0.50 | **<0.001** | 10.85 | 9.97 | 0.88 | **<0.001** | **0.013** |
| Right Lower Limb Fat (kg) | 3.46 | 3.32 | 0.14 | **0.008** | 3.47 | 3.33 | 0.14 | **0.017** | 0.979 |
| Left Lower Limb Fat (kg) | 3.44 | 3.29 | 0.15 | **0.009** | 3.45 | 3.33 | 0.12 | **0.028** | 0.693 |
| Basal Metabolic Rate (kcal) | 1265.70 | 1273.65 | -7.95 | 0.074 | 1284.74 | 1266.79 | 17.95 | **0.005** | **0.001** |

**Table S4**: Nutrient intake changes of two study groups before and after intervention

| **Nutrients** | **MICT Group** | | | | **MICT+MR Group** | | | | ***p* _delta_** |
| --- | --- | --- | --- | --- | --- | --- | --- | --- | --- |
|  | **Baseline** | **Intervention Period** | **Δ_baseline-post_** | ***p*** | **Baseline** | **Intervention Period** | **Δ_baseline-post_** | ***p*** |  |
| Dinner - Energy (kcal) | 407.45 | 518.78 | -111.34 | **0.005** | 401.75 | 263.76 | 137.99 | **0.013** | **<0.001** |
| Dinner - Carbohydrates (g) | 46.74 | 63.54 | -16.80 | **0.005** | 47.37 | 31.01 | 16.36 | **0.017** | **<0.001** |
| Dinner - Fat (g) | 16.58 | 19.56 | -2.98 | 0.149 | 15.74 | 9.23 | 6.51 | **0.029** | **0.007** |
| Dinner - Protein (g) | 17.82 | 22.16 | -4.34 | **0.041** | 17.66 | 14.16 | 3.50 | 0.092 | **0.008** |
| Total Day - Energy (kcal) | 1400.17 | 1390.83 | 9.33 | 0.909 | 1461.57 | 1058.82 | 402.76 | **0.002** | **0.007** |
| Total Day - Carbohydrates (g) | 160.03 | 164.97 | -4.94 | 0.601 | 194.58 | 127.78 | 66.80 | **0.003** | **0.002** |
| Total Day - Fat (g) | 60.38 | 58.09 | 2.29 | 0.590 | 49.07 | 38.85 | 10.23 | 0.058 | 0.232 |
| Total Day - Protein (g) | 54.16 | 52.05 | 2.11 | 0.699 | 60.40 | 49.53 | 10.88 | **0.026** | 0.221 |
| Dietary Fiber (g) | 6.57 | 6.82 | -0.25 | 0.705 | 5.48 | 6.64 | -1.16 | 0.144 | 0.369 |
| Vitamin D (μg) | 1.72 | 1.92 | -0.20 | 0.470 | 1.69 | 2.07 | -0.38 | 0.357 | 0.713 |
| Vitamin B12 (μg) | 1.18 | 1.44 | -0.27 | 0.406 | 0.39 | 0.30 | 0.09 | 0.191 | 0.280 |
| Vitamin B6 (mg) | 0.38 | 0.44 | -0.06 | 0.319 | 0.87 | 0.76 | 0.11 | 0.483 | 0.303 |
| Calcium (mg) | 280.96 | 321.17 | -40.21 | 0.244 | 226.33 | 272.68 | -46.36 | 0.256 | 0.906 |
| Iron (mg) | 11.11 | 10.38 | 0.73 | 0.389 | 8.73 | 5.96 | 2.77 | **0.004** | **0.001** |
| Potassium (mg) | 1127.74 | 1196.78 | -69.04 | 0.369 | 1074.82 | 712.02 | 362.80 | **0.004** | **0.002** |
| Sodium (mg) | 2290.10 | 2569.87 | -279.78 | 0.268 | 2265.73 | 1831.75 | 433.98 | 0.327 | 0.152 |
| Purines (mg) | 135.42 | 155.66 | -20.25 | 0.279 | 119.21 | 87.42 | 31.79 | 0.086 | **0.046** |
| Saturated Fat (g) | 7.78 | 7.64 | 0.14 | 0.905 | 6.68 | 3.79 | 2.89 | **0.005** | 0.065 |
| Sugar (g) | 12.69 | 10.25 | 2.44 | 0.282 | 13.60 | 7.14 | 6.45 | **0.013** | 0.221 |

**Table S5:** Macronutrient energy ratios before and after intervention in two study groups

|  | **MICT Group** | | | **MICT+MR Group** | | |
| --- | --- | --- | --- | --- | --- | --- |
|  | **Baseline** | **Intervention Period** | ***p*** | **Baseline** | **Intervention Period** | ***p*** |
| Dinner - Carbohydrates | 47% | 49% | 0.444 | 49% | 46% | 0.464 |
| Dinner - Fat | 36% | 34% | 0.499 | 33% | 30% | 0.383 |
| Dinner - Protein | 17% | 17% | 0.685 | 18% | 23% | **0.036** |
| Total Day - Carbohydrates | 46% | 48% | 0.265 | 53% | 49% | 0.084 |
| Total Day - Fat | 39% | 37% | 0.287 | 30% | 33% | 0.393 |
| Total Day - Protein | 15% | 15% | 0.686 | 17% | 19% | **0.053** |
